# Knowledge, attitude, and perception of Arab medical students towards artificial intelligence in medicine and radiology: A multi-national cross-sectional study

**DOI:** 10.1007/s00330-023-10509-2

**Published:** 2023-12-27

**Authors:** Ahmed Hafez Allam, Nael Kamel Eltewacy, Yasmeen Jamal Alabdallat, Tarek A. Owais, Saif Salman, Mahmoud A. Ebada, for the EARG Group, Hajar Alkokhiya Aldare, Mohammed Amir Rais, Moath Salem, Jaafar D. Al-Dabagh, Monzer Abdulatif Alhassan, Marah M. Hanjul, Tayba Abdulrahman Mugibel, Sara Hamada Motawea, Mirna Hussein, Omar Saeed Anas, Nacer Mohamed Amine, Moath Ahmed Almekhlafi, Muna Ali Mugibel, Eman Salem Barhoom, Haroun Neiroukh, Raghad Shweiki, Mohammad Khalaf Balaw, Mohmmad Ahmad Al-Slehat, Zaynab Roze, Maram A. Sadeq, Fathia Mokhtar, Noora Mahdi Babiker, Rami Abd Al-Ati, Huda Adel Alhoudairi, Mohammed Omran Attayeb, Abdulrhman Abdulhadi, Abdulghani Arja, Abdulkareem Muhammad Wardeh, Dana Nabil Alakhrass, Souad Alkanj

**Affiliations:** 1 Faculty of Medicine, Menoufia University, Shebin El-Kom, Menoufia Egypt; 2 Eltewacy Arab Research Group, Cairo, Egypt; 3 Faculty of Pharmacy, Beni-Suef University, Beni-Suef, Egypt; 4 Faculty of Medicine, Hashemite University, Zarqa, Jordan; 5 Mayo Clinic College of Medicine, Jacksonville, FL USA; 6 Faculty of Medicine, Zagazig University, Zagazig, El-Sharkia Egypt; 7 Egyptian Fellowship of Neurology, Nasr City Hospital for Health Insurance, Nasr City, Cairo Egypt

**Keywords:** Artificial intelligence, AI education, AI perception, Students, Medical, Radiology training

## Abstract

**Objectives:**

We aimed to assess undergraduate medical students’ knowledge, attitude, and perception regarding artificial intelligence (AI) in medicine.

**Methods:**

A multi-national, multi-center cross-sectional study was conducted from March to April 2022, targeting undergraduate medical students in nine Arab countries. The study utilized a web-based questionnaire, with data collection carried out with the help of national leaders and local collaborators. Logistic regression analysis was performed to identify predictors of knowledge, attitude, and perception among the participants. Additionally, cluster analysis was employed to identify shared patterns within their responses.

**Results:**

Of the 4492 students surveyed, 92.4% had not received formal AI training. Regarding AI and deep learning (DL), 87.1% exhibited a low level of knowledge. Most students (84.9%) believed AI would revolutionize medicine and radiology, with 48.9% agreeing that it could reduce the need for radiologists. Students with high/moderate AI knowledge and training had higher odds of agreeing to endorse AI replacing radiologists, reducing their numbers, and being less likely to consider radiology as a career compared to those with low knowledge/no AI training. Additionally, the majority agreed that AI would aid in the automated detection and diagnosis of pathologies.

**Conclusions:**

Arab medical students exhibit a notable deficit in their knowledge and training pertaining to AI. Despite this, they hold a positive perception of AI implementation in medicine and radiology, demonstrating a clear understanding of its significance for the healthcare system and medical curriculum.

**Clinical relevance statement:**

This study highlights the need for widespread education and training in artificial intelligence for Arab medical students, indicating its significance for healthcare systems and medical curricula.

**Key Points:**

• *Arab medical students demonstrate a significant knowledge and training gap when it comes to using AI in the fields of medicine and radiology*.

• *Arab medical students recognize the importance of integrating AI into the medical curriculum. Students with a deeper understanding of AI were more likely to agree that all medical students should receive AI education. However, those with previous AI training were less supportive of this idea*.

• *Students with moderate/high AI knowledge and training displayed increased odds of agreeing that AI has the potential to replace radiologists, reduce the demand for their services, and were less inclined to pursue a career in radiology, when compared to students with low knowledge/no AI training*.

**Supplementary Information:**

The online version contains supplementary material available at 10.1007/s00330-023-10509-2.

## Introduction

Artificial intelligence (AI) is a field of computer science that mimics human intelligence in learning and solving problems. One subfield of AI, machine learning (ML), focuses on developing algorithms capable of improving accuracy through pattern recognition and data analysis [1–3]. Deep learning (DL), which falls under the umbrella of ML, has garnered significant attention in the healthcare sector. DL utilizes artificial neural networks to process and analyze large volumes of data, making it especially valuable for image processing, analysis, and even aiding in robotic surgeries [4, 5]. In the medical field, AI research encompasses a broad spectrum of applications. This includes collecting and interpreting healthcare data, imaging techniques, and extending AI’s capabilities to therapeutic and surgical approaches. Additionally, AI plays a vital role in providing timely warnings to patients and healthcare professionals when necessary [6].

The application of AI in radiology has significant implications, as FDA-approved AI-based algorithms have demonstrated remarkable accuracy in detecting specific diseases, comparable to human experts in terms of specificity and sensitivity [7]. However, the rapid technological advancements enabling the growth of AI have sparked discussions surrounding the future of diagnostic and interventional radiology, giving rise to concerns about the potential impact on the practice’s long-term viability [8]. Over the past decade, AI and ML have been the subject of intense debate within the field of radiology, as evidenced by the publication of more than 5000 articles between 2018 and 2023, according to a search conducted on PubMed as of March 25, 2023.

Consequently, these technological advancements have generated a substantial knowledge base and diverse perspectives on AI’s role in medicine. Surveys have revealed that radiologists’ attitudes towards AI range from enthusiastic acceptance to skepticism, primarily driven by fears of being displaced by technology [9, 10]. Notably, the popularity of radiology as a career choice among medical graduates in the USA has declined since the 1990s [11, 12]. Contrary to concerns about replacement, the European Society of Radiology asserts that AI will not replace radiologists but rather enhance their value and improve the field as a whole [13]. In response, radiologists must proactively learn about AI and its applications and collaborate with AI researchers to optimize patient care. Furthermore, the impact of AI extends beyond radiology and will similarly influence other healthcare professions, including pathology, cardiology, and others [13, 14].

Given the recent advancements in AI within the healthcare system, it is increasingly evident that doctors and medical students require comprehensive education in AI. Consequently, raising awareness of AI among future healthcare professionals is crucial to guide their career choices. This topic has received significant attention in Europe, Canada, and the USA; however, it remains relatively understudied in the Middle East and Arab countries. Limited information is available regarding Arab medical students’ awareness and perspectives towards AI and DL in medicine and radiology, as well as the factors influencing their knowledge and attitudes, such as demographics, academic performance, technological proficiency, and previous AI training. Furthermore, it is crucial to investigate their opinions regarding integrating AI into medical school curricula and explore potential differences between Arab and foreign medical students’ attitudes and perceptions.

We aimed to evaluate students’ knowledge, attitude, and perception concerning the utilization of AI in medicine, with a specific focus on its application in radiology. Furthermore, we sought to identify variations in perceptions and attitudes among different student groups. Through this investigation, we aimed to gain insights into the thoughts and sentiments of these students regarding AI in medicine, as well as to determine the potential utility of incorporating the study of AI applications as a compulsory component within their educational curriculum.

## Methods

### Design

We conducted a multi-national, multi-center cross-sectional study among undergraduate medical students in nine countries in the Middle East and North Africa (MENA) region (Libya, Egypt, Iraq, Jordan, Syria, Sudan, Algeria, Palestine, and Yemen) between March 1, 2022, and April 13, 2022, using an online self-administered questionnaire. All undergraduate students were included. There were no exclusion criteria regarding age or gender. The Strengthening the Reporting of Observational Studies in Epidemiology (STROBE) Checklist was followed in the conduct and reporting of the current article.

### Sampling

We adopted the convenience sampling method in our study. The Raosoft sample size calculator was used to estimate the sample size [15]. With a 5% margin of error, a 95% confidence level, and a 50% response distribution (according to a study in Saudi Arabia which found that approximately 50% of the students believed they had a good understanding of AI; however, when knowledge of AI was tested, only 22% of the questions were answered correctly [16]), the sample size was calculated to be at least 382 students per country.

### Questionnaire development and validation

The questionnaire was developed using frequently asked questions from previously published national surveys in Canada, the UK, Croatia, the USA, and Germany [17–21]. Experts in the fields of AI and radiology revised each question in terms of relevance, comprehensiveness, and clarity, and some details were improved according to their comments. The questionnaire was translated into Arabic by two bilingual translators, and then the Arabic version was translated back into English by two different translators. The new English version was compared to the original one until a final version was agreed upon. The questionnaire was distributed in both languages; each participant could select his/her preferred language. We also conducted a pilot sampling for both Arabic and English versions with a total of 265 responses that were not included in the analysis to assess Cronbach’s alpha for each domain of the questionnaire.

The questionnaire included four sections:

1. Socio-demographic data: including gender, country, residence (urban/rural), university, grade, and if they are proficient in using modern technology.
2. Knowledge about AI and DL: consisted of 10 questions about the basic principles of AI, its limitations, and whether they are familiar with the terminology related to AI. *Cronbach’s alpha for this section was 0.75.*
3. Attitudes towards AI and DL: consisted of 18 questions assessing their feelings and perspectives towards AI and DL in medicine and radiology. *Cronbach’s alpha for this section was 0.81.*
4. Perception regarding AI: consisted of four questions: one assessing whether they accept working alongside AI in a certain clinical workflow, and three asking about AI’s potential applications in radiology practice. *Cronbach’s alpha for this section was 0.66.*

Finally, four more questions were added. One about which specialties students think would be impacted the earliest and the most by AI, and another three questions about whether they had AI training.

The confirmatory factor analysis model demonstrated an acceptable fit for the data. The model was tested against a baseline model, which revealed a statistically significant difference between the two models. The comparative fit index (CFI) and Tucker-Lewis index (TLI) values were 0.460 and 0.417, respectively, suggesting adequate model fit. The root mean square error of approximation (RMSEA) was 0.123, with a 90% confidence interval between 0.118 and 0.129, indicating a reasonable fit. The loading factors for all three sections of the questionnaire are demonstrated in Table S1 (Supplementary material).

The Arabic and English versions of the questionnaire are illustrated in the Supplementary material.

### Data collection

To ensure the quality of the data collection process, we designated a national leader responsible for their country’s data collection process and obtaining ethical approval. We taught them about the nature of the study and the data collection strategy. Each national leader recruited two to five collaborators between January 1, 2022, and February 10, 2022, to help collect the required sample.

Online Google Forms were used for data collection. There were no duplicates since each respondent was only permitted to fill out the questionnaire once via activating the limit to one response option in the settings list, where you can only answer the survey through your email once. Data from the online questionnaires were automatically collected and kept in an Excel spreadsheet. Each collaborator could only access their replies; however, the central investigator could access all responses throughout the country. Arabic answers were translated into English and merged with the English responses in a single Excel spreadsheet for analysis.

### Ethical considerations

We obtained ethical approval from the Ethics Committee in six countries before starting the data collection process. In addition, written consent was obtained from the participants after a detailed explanation of the study before filling out the questionnaire, emphasizing their confidentiality and the complete preservation of their data.

### Data analysis

Descriptive statistics and regression analyses were performed using R Statistical Software (v4.1.3; R Core Team 2022). Simple descriptive statistics were used to represent the attitude and perceptions of the students using frequencies with percentages. The knowledge section was rated as high (>80% correct answers), moderate (60 to 80% correct answers), and low knowledge level (<60% correct answers). Univariate and multivariate logistic regression analyses were used to assess students’ knowledge, attitude, and perception predictors (demographics including gender, grade, university, place of living, technology experience, and previous AI training). We also used the Hosmer–Lemeshow test to assess the goodness of fit for the regression models.

K-means cluster analyses were performed using Python 3.10.6 to find the patterns of students’ attitudes and perceptions and to see which participants have which kind of perspectives. We standardized the variables using the StandardScaler function, which modifies the data distribution to have a mean of 0 and a standard deviation of 1. This step ensured that each variable had equal weight in the clustering process. The optimal number of clusters was determined using the silhouette score method, which evaluates the clustering quality based on the similarity of objects within a cluster and the dissimilarity between clusters. The higher the score, the better fit the cluster analysis. A chi-square *p* value was used to assess the statistical significance of the variables in the clusters.

## Results

### Demographic data

The study sample comprised 4492 medical students from nine countries. In all countries except Yemen, females outnumbered males, with 2768 (61.6%) female participants. Most participants studied in public universities (*n* = 3877, 86.3%) and lived in urban zones (*n* = 3486, 77.6%). About 19.2% of the participants (*n* = 864) were in their third year at medical school. Furthermore, 1784 participants (39.7%) consider themselves neutral regarding tech-savviness. The demographics of the included participants are shown in Table 1.

**Table 1:** Demographic characteristics of participating Arab medical students

	Levels	Algeria	Egypt	Iraq	Jordan	Libya	Palestine	Sudan	Syria	Yemen	Total
		*N* = 437	*N* = 563	*N* = 533	*N* = 508	*N* = 730	*N* = 388	*N* = 474	*N* = 475	*N* = 384	*N* = 4492
Gender	Female	256 (58.6)	345 (61.3)	327 (61.4)	271 (53.3)	600 (82.2)	248 (63.9)	318 (67.1)	219 (46.1)	184 (47.9)	2768 (61.6)
	Male	181 (41.4)	218 (38.7)	206 (38.6)	237 (46.7)	130 (17.8)	140 (36.1)	156 (32.9)	256 (53.9)	200 (52.1)	1724 (38.4)
University	Governmental	427 (97.7)	525 (93.3)	495 (92.9)	504 (99.2)	698 (95.6)	135 (34.8)	405 (85.4)	342 (72.0)	346 (90.1)	3877 (86.3)
	International	10 (2.3)	3 (0.5)	5 (0.9)	3 (0.6)	22 (3.0)	17 (4.4)	3 (0.6)	2 (0.4)	4 (1.0)	69 (1.5)
	Private	-	35 (6.2)	33 (6.2)	1 (0.2)	10 (1.4)	236 (60.8)	66 (13.9)	131 (27.6)	34 (8.9)	546 (12.2)
Living zone	Rural	73 (16.7)	211 (37.5)	78 (14.6)	93 (18.3)	158 (21.6)	133 (34.3)	120 (25.3)	87 (18.3)	53 (13.8)	1006 (22.4)
	Urban	364 (83.3)	352 (62.5)	455 (85.4)	415 (81.7)	572 (78.4)	255 (65.7)	354 (74.7)	388 (81.7)	331 (86.2)	3486 (77.6)
Grade	1	33 (7.6)	31 (5.5)	161 (30.2)	85 (16.7)	261 (35.8)	88 (22.7)	51 (10.8)	13 (2.7)	51 (13.3)	774 (17.2)
	2	43 (9.8)	48 (8.5)	174 (32.6)	82 (16.1)	94 (12.9)	147 (37.9)	76 (16.0)	36 (7.6)	30 (7.8)	730 (16.3)
	3	51 (11.7)	72 (12.8)	101 (18.9)	172 (33.9)	108 (14.8)	111 (28.6)	141 (29.7)	66 (13.9)	42 (10.9)	864 (19.2)
	4	67 (15.3)	149 (26.5)	40 (7.5)	78 (15.4)	94 (12.9)	19 (4.9)	73 (15.4)	117 (24.6)	114 (29.7)	751 (16.7)
	5	81 (18.5)	79 (14.0)	26 (4.9)	58 (11.4)	58 (7.9)	8 (2.1)	69 (14.6)	113 (23.8)	36 (9.4)	528 (11.8)
	6	58 (13.3)	142 (25.2)	16 (3.0)	16 (3.1)	47 (6.4)	10 (2.6)	42 (8.9)	68 (14.3)	72 (18.8)	471 (10.5)
	Intern	104 (23.8)	42 (7.5)	15 (2.8)	17 (3.3)	68 (9.3)	5 (1.3)	22 (4.6)	62 (13.1)	39 (10.2)	374 (8.3)
Tech-savviness	1 (Strongly disagree)	100 (22.9)	42 (7.5)	76 (14.3)	25 (4.9)	65 (8.9)	17 (4.4)	39 (8.2)	32 (6.7)	34 (8.9)	430 (9.6)
	2 (Disagree)	108 (24.7)	113 (20.1)	126 (23.6)	78 (15.4)	117 (16.0)	55 (14.2)	104 (21.9)	100 (21.1)	73 (19.0)	874 (19.5)
	3 (Neutral)	157 (35.9)	215 (38.2)	206 (38.6)	201 (39.6)	293 (40.1)	177 (45.6)	201 (42.4)	185 (38.9)	149 (38.8)	1784 (39.7)
	4 (Agree)	56 (12.8)	140 (24.9)	69 (12.9)	148 (29.1)	176 (24.1)	100 (25.8)	82 (17.3)	98 (20.6)	81 (21.1)	950 (21.1)
	5 (Strongly agree)	16 (3.7)	53 (9.4)	56 (10.5)	56 (11.0)	79 (10.8)	39 (10.1)	48 (10.1)	60 (12.6)	47 (12.2)	454 (10.1)

Data are presented as frequency (%)

### Knowledge

**Fig. 1:**
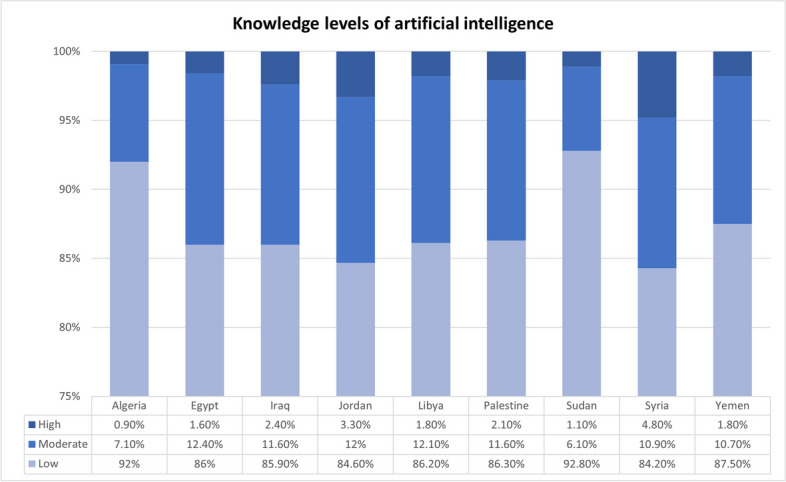
Assessment of knowledge levels of artificial intelligence among Arab medical students

Most students (*n* = 3914, 87.1%) had a low level of knowledge regarding AI. Moreover, 479 students (10.6%) and 99 students (2.2%) had moderate and high levels of knowledge, respectively. 83.7% of the students (*n* = 3762) had a low level of knowledge regarding DL. Sudan had the highest percentage of students (*n* = 440, 92.8%) with a low knowledge of basic AI computational principles, terminology, limitations, and DL. In contrast, the highest number of students with a high level of knowledge was reported among Syrians (*n* = 23, 4.8%), as shown in Fig. 1.

### Attitude

#### Artificial intelligence and deep learning

Concerning the feelings and attitudes towards AI and DL in medicine and radiology, 1958 participants strongly agree that AI will revolutionize radiology (43.6%). Moreover, 1593 responses were neutral concerning human radiologist replacement in the future (35.5%); meanwhile, 1620 students (36.1%) agreed that it would reduce the number of needed radiologists. 38.7% of the responses (*n* = 1738) were neutral regarding students’ likelihood of considering a career in radiology given the advancement of AI (Table S2, Supplementary material).

#### Artificial intelligence and medical curriculum

The majority of participants strongly agreed that all medical students should receive teaching in AI (*n* = 1950, 43.4%) and that it will be beneficial for their career (*n* = 1850, 41.2%), as shown in Table S3 (Supplementary material).

### Artificial intelligence training

92.4% of participants (*n* = 4152) received no teaching or training in AI. Only 142 students (41.8%) of those who received the training received it as part of their curriculum, and 111 (32.6%) of those who received the training rated their satisfaction to be 3 out of 5 (neutral).

### Specialties affected by artificial intelligence

**Fig. 2:**
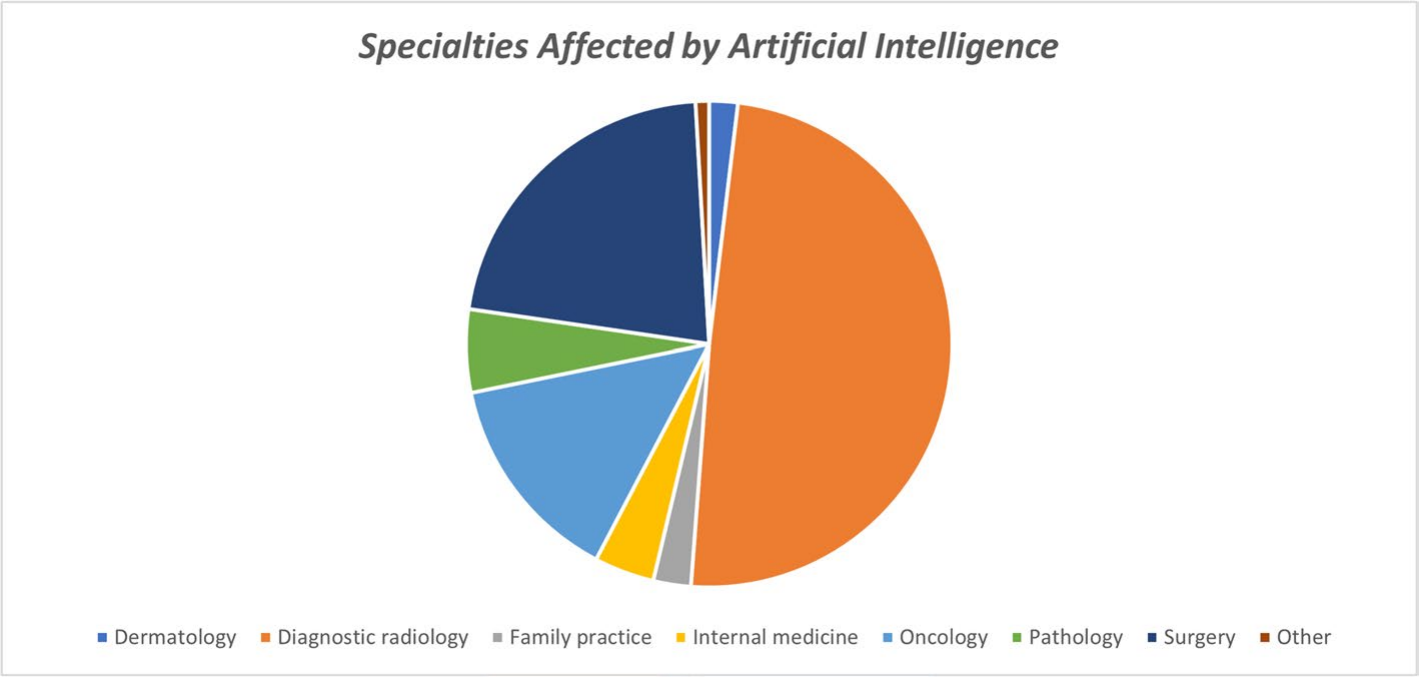
Arab medical students’ perceived impact of artificial intelligence on different medical specialties

Diagnostic radiology was reported to be the most affected specialty at the early stages of developing AI applications (*n* = 2214, 49.3%), followed by surgery and oncology, as shown in Fig. 2.

### Regression analysis

#### Knowledge levels (high/moderate vs. low)

The multivariate logistic regression analysis showed that students studying in a private university (adjusted odds ratio (AOR): 1.55; 95%CI: 1.18–2.03; *p* = 0.001), being neutral (AOR: 3.45; 95%CI: 1.87–7.11; *p* < 0.001), “agreed” (AOR: 10.14; 95%CI: 5.53–20.89; *p* < 0.001) or “strongly agreed” (AOR: 25.17; 95%CI: 13.52–52.35; *p* < 0.001) that they were tech-savvy, and having AI training (AOR: 7.63; 95%CI: 5.86–9.94; *p* < 0.001) were found to be independently associated with increased odds of getting a high/moderate level of knowledge (Table 2). The test statistic of the Hosmer–Lemeshow test was − 30,930 with 8 degrees of freedom, and the *p* value was greater than 0.5, indicating a good fit for the model.

**Table 2:** Multivariate logistic regression analysis showing the predictors of knowledge (high/moderate vs. low) among Arab medical students

Multivariate logistic regression analysis showing the predictors of knowledge levels (high/moderate vs. low)						
Dependent variable	Independent variable	Categories	Low (< 60% of right answers)	High/moderate (> = 60% right answers)	Unadjusted OR (95% confidence interval, *p* value)	Adjusted OR (95% confidence interval, *p* value)
Knowledge levels	Gender	Female^a^	2469 (89.2)	299 (10.8)	-	-
		Male	1445 (83.8)	279 (16.2)	1.59 (1.34–1.90, *p* < 0.001)	1.05 (0.86–1.29, *p* = 0.627)
	University	Governmental^a^	3422 (88.3)	455 (11.7)	-	-
		International	55 (79.7)	14 (20.3)	1.91 (1.02–3.37, *p* = 0.032)	1.14 (0.54–2.28, *p* = 0.711)
		Private	437 (80.0)	109 (20.0)	1.88 (1.48–2.36, *p* < 0.001)	1.55 (1.18–2.03, *p* = 0.001)
	Living zone	Rural^a^	893 (88.8)	113 (11.2)	-	-
		Urban	3021 (86.7)	465 (13.3)	1.22 (0.98–1.52, *p* = 0.079)	1.03 (0.81–1.33, *p* = 0.791)
	Grade	1^a^	689 (89.0)	85 (11.0)	-	-
		2	635 (87.0)	95 (13.0)	1.21 (0.89–1.66, *p* = 0.226)	1.10 (0.77–1.57, *p* = 0.606)
		3	758 (87.7)	106 (12.3)	1.13 (0.84–1.54, *p* = 0.418)	1.05 (0.75–1.48, *p* = 0.781)
		4	646 (86.0)	105 (14.0)	1.32 (0.97–1.79, *p* = 0.077)	1.18 (0.83–1.67, *p* = 0.356)
		5	460 (87.1)	68 (12.9)	1.20 (0.85–1.68, *p* = 0.297)	1.15 (0.78–1.69, *p* = 0.468)
		6	417 (88.5)	54 (11.5)	1.05 (0.73–1.50, *p* = 0.793)	1.11 (0.74–1.67, *p* = 0.601)
		Intern	309 (82.6)	65 (17.4)	1.71 (1.20–2.42, *p* = 0.003)	1.44 (0.96–2.14, *p* = 0.076)
	Tech savviness	Strongly disagree^a^	420 (97.7)	10 (2.3)	-	-
		Disagree	846 (96.8)	28 (3.2)	1.39 (0.69–3.03, *p* = 0.377)	1.46 (0.72–3.21, *p* = 0.321)
		Neutral	1641 (92.0)	143 (8.0)	3.66 (2.01–7.49, *p* < 0.001)	3.45 (1.87–7.11, *p* < 0.001)
		Agree	750 (78.9)	200 (21.1)	11.20 (6.19–22.84, *p* < 0.001)	10.14 (5.53–20.89, *p* < 0.001)
		Strongly agree	257 (56.6)	197 (43.4)	32.19 (17.62–66.07, *p* < 0.001)	25.17 (13.52–52.35, *p* < 0.001)
	Artificial intelligence training	No^a^	3749 (90.3)	403 (9.7)	-	-
		Yes	165 (48.5)	175 (51.5)	9.87 (7.79–12.50, *p* < 0.001)	7.63 (5.86–9.94, *p* < 0.001)

*OR*, odds ratio, ^a^reference category

#### Attitude and perception

We observed that students with high/moderate AI knowledge and training were associated with increased odds of strongly agreeing/agreeing that AI will replace radiologists (AOR = 1.69; 95%CI: 1.35–2.12; *p* < 0.001, AOR = 2.81; 95%CI: 2.12–3.76; *p* < 0.001, respectively) and reduce the number of needed radiologists (AOR = 1.35; 95%CI: 1.05–1.75; *p* = 0.023, AOR = 1.36; 95%CI: 0.97–1.94; *p* = 0.076, respectively) compared to students having low knowledge and no AI training. Similarly, having high/moderate AI knowledge and training were found to be associated with higher odds of agreeing (strongly agree or agree) that students were less likely to consider pursuing a career in radiology (AOR = 1.77; 95%CI: 1.33–2.36; *p* < 0.001, AOR = 1.52; 95%CI: 1.21–1.90; *p* < 0.001, respectively).

Furthermore, our study revealed that clinical year medical students (4th, 5th, 6th year, and interns) were independently associated with lower odds of agreeing (strongly agree or agree) that AI will replace radiologists in the future (AOR = 0.83; 95%CI: 0.72–0.97; *p* = 0.021) compared to academic years students (from 1st to 3rd grade).

On the other hand, our study found that having high/moderate AI knowledge was independently associated with higher odds of strongly agreeing or agreeing that all medical students should receive AI training or teaching (AOR = 2.09; 1.46–3.08; *p* < 0.001). However, students who obtained previous AI training were associated with lower odds of agreeing (strongly agree or agree) with that suggestion (AOR = 0.44; 95%CI: 0.31–0.63, *p* < 0.001) compared to untrained subjects.

The *p* value for the Hosmer–Lemeshow tests for all these regression models was greater than 0.5, indicating a good fit for the model.

### Cluster analysis

Through confirmatory factor analysis and Cronbach alpha’s results, our questionnaire was structured into three distinct sections: knowledge (9 variables), attitude (18 variables), and perception (4 variables). A detailed breakdown of these sections can be found in Table S1 of the Supplementary material. Furthermore, the attitude section was further divided into two subsections: AI and DL attitude (13 variables) and AI and medical curriculum attitude (5 variables). We performed three rounds of K-means cluster analysis on all these sections except the knowledge section to assess the patterns of students’ attitudes and perceptions regarding AI. In our cluster analyses, we used silhouette scores as a guide to determine the optimal number of clusters for each analysis. It happened that the silhouette score indicated two clusters as the optimal choice in all three of our cluster analyses. We did not force two clusters; rather, we selected this number based on the highest silhouette score, which indicates a better fit for the analysis.

#### Artificial intelligence and deep learning attitude

Cluster analysis using the K-means method was conducted on the section pertaining to attitudes towards AI and DL, encompassing 13 variables. The analysis resulted in the identification of two distinct clusters, as presented in Table 3, with a silhouette score of 0.325. The selection of these variables was based on their demonstrated relevance in capturing participants’ sentiments and attitudes towards the integration of AI and DL in medicine and radiology, as previously determined through factor analysis. Through an examination of these variables, our aim was to delve into the participants’ attitudes and emotional responses towards AI and gain a comprehensive understanding of the diverse perspectives within the studied population.

**Table 3:** Cluster analysis of the feelings and attitudes of medical students towards artificial intelligence and deep learning in medicine and radiology

		Overall	Cluster 1	Cluster 2	*p* value
Number		4492	3818 (85%)	674 (15%)	
Gender	Female	2768 (61.6)	2335 (61.2)	433 (64.2)	0.14
	Male	1724 (38.4)	1483 (38.8)	241 (35.8)	
University	Governmental	3877 (86.3)	3282 (86.0)	595 (88.3)	0.272
	International	69 (1.5)	60 (1.6)	9 (1.3)	
	Private	546 (12.2)	476 (12.5)	70 (10.4)	
Living zone	Rural	1006 (22.4)	857 (22.4)	149 (22.1)	0.885
	Urban	3486 (77.6)	2961 (77.6)	525 (77.9)	
Grade	Academic years (1st, 2nd, and 3rd)	2368 (52.7)	2005 (52.5)	363 (53.9)	0.547
	Clinical Years (4th, 5th, 6th, and interns)	2124 (47.3)	1813 (47.5)	311 (46.1)	
Knowledge levels	High/moderate	578 (12.9)	542 (14.2)	36 (5.3)	< 0.001
	Low	3914 (87.1)	3276 (85.8)	638 (94.7)	
Tech-savviness	Agree/strongly agree	1404 (31.3)	1259 (33.0)	145 (21.5)	< 0.001
	Disagree/strongly disagree	1304 (29.0)	1064 (27.9)	240 (35.6)	
	Neutral	1784 (39.7)	1495 (39.2)	289 (42.9)	
Artificial intelligence training	Yes	340 (7.6)	306 (8.0)	34 (5.0)	0.009
	No	4152 (92.4)	3512 (92.0)	640 (95.0)	
Artificial intelligence will revolutionize radiology	Disagree/strongly disagree	86 (1.9)	80 (2.1)	6 (0.9)	< 0.001
	Agree/strongly agree	3814 (84.9)	3541 (92.7)	273 (40.5)	
	Neutral	592 (13.2)	197 (5.2)	395 (58.6)	
Artificial intelligence will revolutionize medicine in general	Disagree/strongly disagree	132 (2.9)	118 (3.1)	14 (2.1)	< 0.001
	Agree/strongly agree	3815 (84.9)	3527 (92.4)	288 (42.7)	
	Neutral	545 (12.1)	173 (4.5)	372 (55.2)	
The human radiologist will be replaced in the foreseeable future	Disagree/strongly disagree	1793 (39.9)	1624 (42.5)	169 (25.1)	< 0.001
	Agree/strongly agree	1106 (24.6)	1021 (26.7)	85 (12.6)	
	Neutral	1593 (35.5)	1173 (30.7)	420 (62.3)	
The human non interventional physician will be replaced in the foreseeable future	Disagree/strongly disagree	2720 (60.6)	2470 (64.7)	250 (37.1)	< 0.001
	Agree/strongly agree	645 (14.4)	604 (15.8)	41 (6.1)	
	Neutral	1127 (25.1)	744 (19.5)	383 (56.8)	
In the foreseeable future, all physicians will be replaced	Disagree/strongly disagree	3289 (73.2)	2961 (77.6)	328 (48.7)	< 0.001
	Agree/strongly agree	474 (10.6)	442 (11.6)	32 (4.7)	
	Neutral	729 (16.2)	415 (10.9)	314 (46.6)	
These developments frighten me	Disagree/strongly disagree	1274 (28.4)	1191 (31.2)	83 (12.3)	< 0.001
	Agree/strongly agree	1770 (39.4)	1548 (40.5)	222 (32.9)	
	Neutral	1448 (32.2)	1079 (28.3)	369 (54.7)	
These developments make radiology more exciting to me	Disagree/strongly disagree	574 (12.8)	512 (13.4)	62 (9.2)	< 0.001
	Agree/strongly agree	2561 (57.0)	2447 (64.1)	114 (16.9)	
	Neutral	1357 (30.2)	859 (22.5)	498 (73.9)	
These developments make medicine in general more exciting to me	Disagree/strongly disagree	518 (11.5)	461 (12.1)	57 (8.5)	< 0.001
	Agree/strongly agree	2825 (62.9)	2677 (70.1)	148 (22.0)	
	Neutral	1149 (25.6)	680 (17.8)	469 (69.6)	
Artificial intelligence will never make the human physician expendable	Disagree/strongly disagree	470 (10.5)	432 (11.3)	38 (5.6)	< 0.001
	Agree/strongly agree	3036 (67.6)	2786 (73.0)	250 (37.1)	
	Neutral	986 (22.0)	600 (15.7)	386 (57.3)	
The impact of artificial intelligence alone will reduce the number of radiologists that are needed	Disagree/strongly disagree	792 (17.6)	735 (19.3)	57 (8.5)	< 0.001
	Agree/strongly agree	2194 (48.8)	2050 (53.7)	144 (21.4)	
	Neutral	1506 (33.5)	1033 (27.1)	473 (70.2)	
Artificial intelligence will improve radiology	Disagree/strongly disagree	290 (6.5)	266 (7.0)	24 (3.6)	< 0.001
	Agree/strongly agree	3323 (74.0)	3152 (82.6)	171 (25.4)	
	Neutral	879 (19.6)	400 (10.5)	479 (71.1)	
Artificial intelligence will improve medicine in general	Disagree/strongly disagree	218 (4.9)	198 (5.2)	20 (3.0)	< 0.001
	Agree/strongly agree	3587 (79.9)	3384 (88.6)	203 (30.1)	
	Neutral	687 (15.3)	236 (6.2)	451 (66.9)	
I am less likely to consider a career in radiology given the advancement of artificial intelligence	Disagree/strongly disagree	1470 (32.7)	1358 (35.6)	112 (16.6)	< 0.001
	Agree/strongly agree	1284 (28.6)	1184 (31.0)	100 (14.8)	
	Neutral	1738 (38.7)	1276 (33.4)	462 (68.5)	

Data are presented as frequency (%). K-means cluster analysis of this section included 13 variables with a silhouette score of 0.325. For further details regarding cluster analysis, please refer to the “Cluster analysis” subsection in the “Results” section

Cluster 1 comprised 3818 students, the majority of whom expressed agreement with the transformative potential of AI in medicine and radiology, finding these advancements to be exciting. However, they also acknowledged apprehension towards AI progress and its potential impact on the need for radiologists, expressing concerns about a decrease in demand. Additionally, cluster 1 participants disagreed with the notion of entirely replacing physicians and radiologists and, instead, showed a greater inclination towards considering a career in radiology.

In contrast, cluster 2 consisted of 674 participants, who similarly acknowledged the potential revolution that AI could bring to medicine and radiology. However, their perspectives were more neutral when it came to the replacement of radiologists and physicians by AI, their personal fears surrounding AI, their interest in pursuing a career in radiology, and the potential decrease in the number of radiologists due to AI.

Notably, cluster 1 displayed a higher level of technological proficiency, possessing more comprehensive knowledge and training in AI compared to cluster 2. No significant variations were observed between the two clusters in terms of gender, university affiliation, academic grade, or residential location.

#### Artificial intelligence and medical curriculum attitude

For the analysis of the AI and medical curriculum attitude section, five relevant variables were employed in the K-means cluster analysis. This analysis generated two distinct clusters, as displayed in Table 4, with a silhouette score of 0.42. The selection of these variables was guided by their demonstrated relevance in evaluating students’ attitudes and perceptions regarding the incorporation of AI into the medical curriculum, as determined through previous factor analysis. By exploring these variables, our objective was to gain valuable insights into the students’ viewpoints concerning the integration of AI within the medical curriculum and their preparedness to engage with AI technologies.

**Table 4:** Cluster analysis of the students’ attitude towards artificial intelligence and medical curriculum

		Overall	Cluster 1	Cluster 2	*p* value
Number		4492	3084 (68.65%)	1408 (31.35%)	
Gender	Female	2768 (61.6)	1893 (61.4)	875 (62.1)	0.649
	Male	1724 (38.4)	1191 (38.6)	533 (37.9)	
University	Governmental	3877 (86.3)	2669 (86.5)	1208 (85.8)	0.735
	International	69 (1.5)	48 (1.6)	21 (1.5)	
	Private	546 (12.2)	367 (11.9)	179 (12.7)	
Living zone	Rural	1006 (22.4)	695 (22.5)	311 (22.1)	0.768
	Urban	3486 (77.6)	2389 (77.5)	1097 (77.9)	
Grade	Academic years (1st, 2nd, and 3rd)	2368 (52.7)	1622 (52.6)	746 (53.0)	0.834
	Clinical Years (4th, 5th, 6th, and interns)	2124 (47.3)	1462 (47.4)	662 (47.0)	
Knowledge levels	High/moderate	578 (12.9)	436 (14.1)	142 (10.1)	< 0.001
	Low	3914 (87.1)	2648 (85.9)	1266 (89.9)	
Tech-savviness	Agree/strongly agree	1404 (31.3)	990 (32.1)	414 (29.4)	< 0.001
	Disagree/strongly disagree	1304 (29.0)	941 (30.5)	363 (25.8)	
	Neutral	1784 (39.7)	1153 (37.4)	631 (44.8)	
Artificial intelligence training	Yes	340 (7.6)	248 (8.0)	92 (6.5)	0.087
	No	4152 (92.4)	2836 (92.0)	1316 (93.5)	
All medical students should receive teaching in artificial intelligence	Disagree/strongly disagree	440 (9.8)	336 (10.9)	104 (7.4)	< 0.001
	Agree/strongly agree	3132 (69.7)	2369 (76.8)	763 (54.2)	
	Neutral	920 (20.5)	379 (12.3)	541 (38.4)	
Teaching in artificial intelligence will be beneficial for my career	Disagree/strongly disagree	380 (8.5)	291 (9.4)	89 (6.3)	< 0.001
	Agree/strongly agree	3183 (70.9)	2361 (76.6)	822 (58.4)	
	Neutral	929 (20.7)	432 (14.0)	497 (35.3)	
At the end of my medical degree, I will.be confident in using basic healthcare artificial intelligence tools if required	Disagree/strongly disagree	1026 (22.8)	950 (30.8)	76 (5.4)	< 0.001
	Agree/strongly agree	2170 (48.3)	1861 (60.3)	309 (21.9)	
	Neutral	1296 (28.9)	273 (8.9)	1023 (72.7)	
At the end of my medical degree, I will have a better understanding of the methods used to assess healthcare artificial intelligence algorithm performance	Disagree/strongly disagree	1243 (27.7)	1138 (36.9)	105 (7.5)	< 0.001
	Agree/strongly agree	1853 (41.3)	1706 (55.3)	147 (10.4)	
	Neutral	1396 (31.1)	240 (7.8)	1156 (82.1)	
Overall, at the end of my medical degree, I feel I will possess the knowledge needed to work with artificial intelligence in routine clinical practice	Disagree/strongly disagree	1258 (28.0)	1123 (36.4)	135 (9.6)	< 0.001
	Agree/strongly agree	1892 (42.1)	1699 (55.1)	193 (13.7)	
	Neutral	1342 (29.9)	262 (8.5)	1080 (76.7)	

Data are presented as frequency (%). K-means cluster analysis of this section included five variables with a silhouette score of 0.42. For further details regarding cluster analysis, please refer to the “Cluster analysis” subsection in the “Results” section

Cluster 1 comprised 3084 participants, who demonstrated a higher level of AI knowledge, technological proficiency (tech-savviness), and experience compared to cluster 2 (*n* = 1408). Both clusters exhibited agreement regarding the importance of incorporating AI education into the medical curriculum, recognizing its potential benefits for their future careers. However, cluster 1 students predominantly expressed confidence, understanding, and knowledge pertaining to the utilization of fundamental AI tools and methods in healthcare, while cluster 2 students maintained a neutral stance on these aspects.

Notably, no significant variations were observed between the two clusters in terms of gender distribution, university affiliation, academic grade, residential location, or AI training.

#### Perception

To investigate the perception of medical students towards the integration of AI in radiology, a K-means cluster analysis was conducted on the perception section, utilizing four pertinent variables. The analysis yielded two distinct clusters, as depicted in Table 5, with a silhouette score of 0.5. These variables were selected based on their demonstrated relevance in capturing the perceptions of medical students regarding the integration of AI in radiology, as determined by prior factor analysis. By examining these variables, our aim was to explore the students’ perspectives on the potential applications and benefits of AI in radiology, as well as their willingness to embrace AI technologies.

**Table 5:** Cluster analysis of the perception of medical students towards artificial intelligence and radiology

		Overall	Cluster 1	Cluster 2	*p* value
Number		4492	3536 (78.72%)	956 (21.28%)	
Gender	Female	2768 (61.6)	2183 (61.7)	585 (61.2)	0.788
	Male	1724 (38.4)	1353 (38.3)	371 (38.8)	
University	Governmental	3877 (86.3)	3035 (85.8)	842 (88.1)	0.172
	International	69 (1.5)	58 (1.6)	11 (1.2)	
	Private	546 (12.2)	443 (12.5)	103 (10.8)	
Living zone	Rural	1006 (22.4)	777 (22.0)	229 (24.0)	0.208
	Urban	3486 (77.6)	2759 (78.0)	727 (76.0)	
Grade	Academic years (1st, 2nd, and 3rd)	2368 (52.7)	1866 (52.8)	502 (52.5)	0.915
	Clinical Years (4th, 5th, 6th, and interns)	2124 (47.3)	1670 (47.2)	454 (47.5)	
Knowledge levels	High/moderate	578 (12.9)	514 (14.5)	64 (6.7)	< 0.001
	Low	3914 (87.1)	3022 (85.5)	892 (93.3)	
Tech-savviness	Agree/strongly agree	1404 (31.3)	1175 (33.2)	229 (24.0)	< 0.001
	Disagree/strongly disagree	1304 (29.0)	945 (26.7)	359 (37.6)	
	Neutral	1784 (39.7)	1416 (40.0)	368 (38.5)	
Artificial intelligence training	Yes	340 (7.6)	292 (8.3)	48 (5.0)	0.001
	No	4152 (92.4)	3244 (91.7)	908 (95.0)	
Would you consider using the following clinical workflow? Patients’ clinical images undergo artificial intelligence analysis. A specialist subsequently reviews both the image and the artificial intelligence findings	Disagree/strongly disagree	330 (7.3)	254 (7.2)	76 (7.9)	< 0.001
	Agree/strongly agree	2820 (62.8)	2405 (68.0)	415 (43.4)	
	Neutral	1342 (29.9)	877 (24.8)	465 (48.6)	
Automated detection of pathologies in imaging exams	Disagree/strongly disagree	227 (5.1)	221 (6.2)	6 (0.6)	< 0.001
	Agree/strongly agree	3341 (74.4)	3132 (88.6)	209 (21.9)	
	Neutral	924 (20.6)	183 (5.2)	741 (77.5)	
Automated diagnosis in imaging exams	Disagree/strongly disagree	568 (12.6)	547 (15.5)	21 (2.2)	< 0.001
	Agree/strongly agree	2648 (58.9)	2585 (73.1)	63 (6.6)	
	Neutral	1276 (28.4)	404 (11.4)	872 (91.2)	
Automated indication of appropriate imaging exams	Disagree/strongly disagree	367 (8.2)	359 (10.2)	8 (0.8)	< 0.001
	Agree/strongly agree	2808 (62.5)	2707 (76.6)	101 (10.6)	
	Neutral	1317 (29.3)	470 (13.3)	847 (88.6)	

Data are presented as frequency (%). K-means cluster analysis of the perception section included four variables with a silhouette score of 0.5. For further details regarding cluster analysis, please refer to the “Cluster analysis” subsection in the “Results” section

Cluster 1 encompassed 3536 students, the majority of whom expressed agreement with the notion of working collaboratively with AI in a specific workflow, involving a review of both medical images and AI-generated findings subsequent to the initial AI analysis. They also acknowledged the potential applications of AI in detecting and diagnosing pathologies, as well as appropriate indications for imaging examinations. In contrast, cluster 2 (*n* = 956) exhibited predominantly neutral responses to these questions.

Cluster 1 comprised a larger proportion of students with advanced AI knowledge, training, and technological experience (tech-savviness) compared to cluster 2. No significant differences were observed between the two clusters in terms of gender distribution, university affiliation, academic grade, or residential location.

## Discussion

Our findings revealed a concerning lack of knowledge regarding AI and DL among the majority of students. This inadequacy is notably higher compared to findings from previous studies. For instance, a study conducted by Santos et al in Germany reported that 52% of students were aware of the ongoing discussion surrounding the utilization of AI in radiology, with 68% indicating their lack of awareness regarding the underlying technologies [18]. Similarly, a study conducted in Saudi Arabia found that approximately 50% of students believed they possessed a good understanding of AI, but when tested on their knowledge, only 22% answered the questions correctly [16]. Furthermore, a study conducted in Brazil demonstrated that 64.3% of students claimed to lack sufficient knowledge regarding the new advancements in AI technologies [22].

The transformative potential of AI in various medical disciplines, including radiology, is already widely recognized. Consistent with this recognition, the majority of students in our cohort expressed agreement with the notion that AI will exert a substantial impact on healthcare. Correspondingly, a study conducted in the UK reported that 88% of students shared the belief that AI would play a crucial role in the realm of healthcare [19]. Moreover, findings from another study indicated that radiologists themselves anticipate significant changes within the radiology field due to AI within a decade, envisioning its potential roles as a secondary reader and workflow optimizer [23].

However, despite the existence of compelling arguments to the contrary, radiologists continue to harbor significant concerns regarding potential career displacement resulting from further AI integration in the medical field [24]. Our study aimed to investigate the attitudes of Arab students towards AI in medicine and radiology, leading to the identification of two distinct clusters. The larger and more knowledgeable cluster expressed apprehension towards AI advancements and held the belief that AI would lead to a decrease in the demand for radiologists. However, they were predominantly opposed to the complete replacement of radiologists by AI and expressed a continued interest in pursuing a career in radiology. In a study conducted in Saudi Arabia by Bin Dahmash et al [16] among participants who ranked radiology as their first, second, or third career choice, 52% disagreed with the notion of radiologists being replaced during their lifetime, while 44.8% agreed that AI would reduce the number of radiologists needed in the future. Gong et al [17] reported that 67.7% of students believed that AI would diminish the demand for radiologists in the future, yet 58.6% disputed the notion of AI replacing radiologists entirely. Furthermore, research conducted on German medical students by Santos et al [18] revealed that 82.9% of participants did not foresee AI eventually replacing radiologists. Additionally, a study investigating the perception, knowledge, and attitude of radiologists and radiology residents towards AI found that 48% displayed an open and proactive attitude towards AI, while 38% expressed fear regarding potential replacement by AI [25].

Our findings indicate that the influence of AI advancements is perceived more negatively by Arab medical students considering a career in radiology compared to their counterparts in the UK [19], Canada [17], and Germany [18]. Furthermore, our regression analysis revealed a noteworthy association between Arab students possessing moderate/high levels of AI knowledge and training and their increased likelihood of agreeing that AI will replace radiologists, reduce the number of required radiologists, and diminish their interest in pursuing a career in radiology when compared to students with low knowledge and no AI training. This observation suggests a potential misunderstanding among these students, highlighting the need for clarification.

Several studies [24, 26, 27] have addressed this concern, demonstrating that AI does not aim to replace radiologists but rather facilitates their work, emphasizing the importance of adhering to established rules and principles to ensure optimal patient outcomes. It is crucial to acknowledge that the role of radiologists extends beyond image interpretation, encompassing collaboration with other physicians in diagnosis and treatment, management of illnesses, performance of image-guided medical interventions (interventional radiology), and various other tasks [25]. Furthermore, it is plausible to attribute this misunderstanding among Arab students to the phenomenon known as the initial overconfidence effect. This effect occurs when individuals possess limited knowledge in a new domain, leading them to develop an unwarranted sense of confidence in their understanding [28]. In our study, the majority of participants had a relatively low level of AI knowledge. However, even those with a somewhat higher level of knowledge may still have encountered limitations due to the aforementioned overconfidence effect. Consequently, this may explain why these students continue to express concerns and fear regarding AI advancements in the field of radiology.

Most participants in our research expressed agreement regarding the necessity of providing comprehensive education in AI to all medical students. Through our analysis, we identified two distinct clusters of responses pertaining to AI and its integration into the medical curriculum. Both clusters shared the viewpoint that AI education is essential and holds significant benefits for the future careers of medical students. However, the larger and more knowledgeable cluster demonstrated a stronger consensus regarding their confidence, understanding, and knowledge surrounding the use of fundamental AI tools in healthcare. These findings are in line with previous studies conducted in the UK, where 89% of medical students believed that incorporating AI education into their curriculum would be advantageous to their careers, with 78% supporting the inclusion of AI training as part of their medical degree [19]. Similarly, a study conducted in Croatia revealed that 89.6% of radiologists and radiology residents supported the integration of AI into medical education and curricula, underscoring the perceived importance of AI adoption within the medical field [21]. Additional research focusing on radiologists and radiology residents demonstrated a strong consensus among them, indicating that AI should be incorporated into residency programs and radiology curricula [23]. However, our research revealed that students who had received prior AI training were less inclined to agree with the suggestion of integrating AI education for all medical students, in contrast to their untrained counterparts. This observation suggests that these trained students, who were exposed to AI tools, may have gained a deeper appreciation for the complexity and challenges associated with these intelligent systems. Consequently, they expressed reservations about widespread AI education, recognizing the intricacies involved despite acknowledging the utility and potential of these technologies.

Our study revealed a predominantly positive perception of AI applications in radiology among Arab students. Specifically, cluster 1, which consisted of a larger group of knowledgeable and trained students in AI, exhibited a stronger agreement towards working alongside AI and recognized the potential benefits of AI in detecting and diagnosing pathologies and appropriate indications in imaging exams. These findings align with a study conducted by Santos et al [18], in which 30 to 43.4% of German students “rather agreed” with the same concept.

More than 92% of the participants reported no prior training in AI, and over 80% had low knowledge in this area. As a result, the validity of their perspectives and attitudes regarding the impact of AI on various healthcare issues may be limited. This underscores a critical point in our data, potentially affecting their representativeness. It is plausible that the opinions of these students regarding AI are shaped by their limited knowledge and understanding.

Furthermore, our regression analysis demonstrated that students studying in private universities, those who exhibited greater proficiency in using modern technology, and those who had received prior AI training were more likely to possess a high/moderate level of AI and DL knowledge. This observation highlights the importance of incorporating AI into medical curricula, particularly in public universities where students often exhibit lower levels of knowledge. By introducing AI into their education, students will have increased exposure to computers and AI tools, enhancing their proficiency in using modern technology and fostering AI literacy among them.

### Strengths and limitations

To our knowledge, this study represents the first comprehensive multi-national investigation of medical students’ knowledge, attitude, and perception regarding AI within the MENA region. Including data from nine Arab countries has allowed for a more extensive assessment of the current landscape. Nevertheless, we acknowledge some limitations to our study, including the use of convenience sampling, which may introduce selection bias and hinder the establishment of causal relationships between the examined independent and dependent variables inherent to the cross-sectional design. Therefore, we encourage future investigations to adopt a longitudinal study design to better elucidate these relationships.

## Conclusion

Arab medical students have considerably poor knowledge and training regarding the use of AI in medicine and radiology. However, they acknowledge AI’s importance for the healthcare system and medical curriculum and have a positive perception towards AI. These findings raise a significant concern that must be addressed immediately to ensure the up-to-date use and practice of modern technology in the medical field.

### Supplementary Information

Below is the link to the electronic supplementary material.

Supplementary file1 (PDF 788 KB)

## Abbreviations

AI: Artificial intelligence
AOR: Adjusted odds ratio
DL: Deep learning
FDA: Food and Drug Administration
MENA: Middle East and North Africa
ML: Machine learning
OR: Odds ratio
STROBE: Strengthening the Reporting of Observational Studies in Epidemiology
UK: United Kingdom

## Declarations

### Guarantor

The scientific guarantor of this publication is Ahmed Hafez Allam.

### Statistics and biometry

No complex statistical methods were necessary for this paper.

### Study subjects or cohorts overlap

No study subjects or cohorts have been previously reported.

### Methodology

• Multi-center study

• Cross-sectional study
