# Supplementary material for: Knowledge, attitude, and perception of Arab medical students towards artificial intelligence in medicine and radiology: A multi-national cross-sectional study
Source: Eur Radiol. 2023 Dec 27;34(7):1–14. doi: 10.1007/s00330-023-10509-2 (PMC11213794; doi:10.1007/s00330-023-10509-2)
Supplement: Supplementary file 1 — Supplementary file1 (PDF 788 KB) [file 330_2023_10509_MOESM1_ESM.pdf]

Knowledge, attitude, and perception of Arab medical students towards artificial intelligence in medicine and radiology: A multi-national cross-sectional study

Electronic Supplementary Material

Table S1. Factor loading for knowledge, attitude, and perception sections.

Questions	Knowledge	Attitude	Perception
I have an understanding of the basic computational principles of artificial intelligence	0.733		
I am comfortable with the nomenclature related to artificial intelligence	0.746		
I have an understanding of the limitations of artificial intelligence	0.762		
Were you already aware of these topics in radiology?	0.332		
Do you personally have a basic understanding of the technologies used in these topics?	0.338		
Deep learning is a class of machine learning algorithms that use multiple layers of neural networks.	0.419		
Deep learning methods learn directly from data, without the need for manual feature extraction.	0.336		
The application of deep learning in radiology requires large databases of labeled medical images.	0.384		
Deep learning systems are often opaque: it can be difficult to delineate the underlying "thought process".	0.366		
Artificial intelligence will revolutionize radiology		0.655	
Artificial intelligence will revolutionize medicine in general		0.545	
The human radiologist will be replaced in the foreseeable future		0.348	
The human non interventional physician will be replaced in the foreseeable future		0.348	
In the foreseeable future, all physicians will be replaced		0.319	
These developments frighten me		-0.069	
These developments make radiology more exciting to me		0.705	
These developments make medicine in general more exciting to me		0.791	
Artificial intelligence will never make the human physician expendable		0.234	
The impact of artificial intelligence alone will reduce the number of radiologists that are needed		0.374	
Artificial intelligence will improve radiology		0.654	
Artificial intelligence will improve medicine in general		0.77	
I am less likely to consider a career in radiology given the advancement of artificial intelligence		0.223	
All medical students should receive teaching in artificial intelligence		0.341	
Teaching in artificial intelligence will be beneficial for my career		0.417	
At the end of my medical degree, I will.be confident in using basic healthcare artificial intelligence tools if required		0.366	

At the end of my medical degree, I will have a better understanding of the methods used to assess healthcare artificial intelligence algorithm performance	0.335	
Overall, at the end of my medical degree, I feel I will possess the knowledge needed to work with artificial intelligence in routine clinical practice	0.325	
Would you consider using the following clinical workflow? Patients' clinical images undergo artificial intelligence analysis. A specialist subsequently reviews both the image and the artificial intelligence findings.		0.297
Automated detection of pathologies in imaging exams		0.699
Automated diagnosis in imaging exams		0.617
Automated indication of appropriate imaging exams		0.673

Table S2. Feelings and attitudes towards artificial intelligence and deep learning in medicine and radiology

		Algeria	Egypt	Iraq	Jordan	Libya	Palestine	Sudan	Syria	Yemen	Total
Artificial intelligence will revolutionize radiology	Agree	190 (43.5)	243 (43.2)	217 (40.7)	215 (42.3)	275 (37.7)	182 (46.9)	200 (42.2)	187 (39.4)	147 (38.3)	1856 (41.3)
	Disagree	3 (0.7)	8 (1.4)	9 (1.7)	2 (0.4)	16 (2.2)	6 (1.5)	9 (1.9)	6 (1.3)	7 (1.8)	66 (1.5)
	Neutral	43 (9.8)	73 (13.0)	89 (16.7)	61 (12.0)	108 (14.8)	40 (10.3)	67 (14.1)	66 (13.9)	45 (11.7)	592 (13.2)
	Strongly agree	200 (45.8)	237 (42.1)	212 (39.8)	229 (45.1)	328 (44.9)	159 (41.0)	197 (41.6)	214 (45.1)	182 (47.4)	1958 (43.6)
	Strongly disagree	1 (0.2)	2 (0.4)	6 (1.1)	1 (0.2)	3 (0.4)	1 (0.3)	1 (0.2)	2 (0.4)	3 (0.8)	20 (0.4)
Artificial intelligence will revolutionize medicine in general	Agree	197 (45.1)	231 (41.0)	198 (37.1)	206 (40.6)	263 (36.0)	169 (43.6)	194 (40.9)	199 (41.9)	150 (39.1)	1807 (40.2)
	Disagree	5 (1.1)	17 (3.0)	14 (2.6)	4 (0.8)	21 (2.9)	9 (2.3)	11 (2.3)	13 (2.7)	10 (2.6)	104 (2.3)
	Neutral	44 (10.1)	61 (10.8)	75 (14.1)	64 (12.6)	104 (14.2)	40 (10.3)	60 (12.7)	57 (12.0)	40 (10.4)	545 (12.1)
	Strongly agree	190 (43.5)	252 (44.8)	240 (45.0)	231 (45.5)	336 (46.0)	169 (43.6)	206 (43.5)	205 (43.2)	179 (46.6)	2008 (44.7)
	Strongly disagree	1 (0.2)	2 (0.4)	6 (1.1)	3 (0.6)	6 (0.8)	1 (0.3)	3 (0.6)	1 (0.2)	5 (1.3)	28 (0.6)
The human radiologist will be replaced in the foreseeable future	Agree	38 (8.7)	96 (17.1)	80 (15.0)	82 (16.1)	121 (16.6)	63 (16.2)	98 (20.7)	81 (17.1)	75 (19.5)	734 (16.3)
	Disagree	190 (43.5)	185 (32.9)	135 (25.3)	141 (27.8)	224 (30.7)	121 (31.2)	130 (27.4)	173 (36.4)	117 (30.5)	1416 (31.5)
	Neutral	129 (29.5)	194 (34.5)	221 (41.5)	195 (38.4)	261 (35.8)	151 (38.9)	186 (39.2)	136 (28.6)	120 (31.2)	1593 (35.5)
	Strongly agree	18 (4.1)	47 (8.3)	50 (9.4)	37 (7.3)	64 (8.8)	28 (7.2)	43 (9.1)	49 (10.3)	36 (9.4)	372 (8.3)
	Strongly disagree	62 (14.2)	41 (7.3)	47 (8.8)	53 (10.4)	60 (8.2)	25 (6.4)	17 (3.6)	36 (7.6)	36 (9.4)	377 (8.4)
The human non interventional physician will be replaced in the foreseeable future	Agree	32 (7.3)	52 (9.2)	44 (8.3)	43 (8.5)	51 (7.0)	45 (11.6)	57 (12.0)	40 (8.4)	35 (9.1)	399 (8.9)
	Disagree	202 (46.2)	235 (41.7)	200 (37.5)	184 (36.2)	325 (44.5)	160 (41.2)	191 (40.3)	202 (42.5)	142 (37.0)	1841 (41.0)
	Neutral	108 (24.7)	126 (22.4)	132 (24.8)	134 (26.4)	190 (26.0)	106 (27.3)	137 (28.9)	107 (22.5)	87 (22.7)	1127 (25.1)

	Strongly agree	14 (3.2)	28 (5.0)	40 (7.5)	28 (5.5)	41 (5.6)	12 (3.1)	27 (5.7)	32 (6.7)	24 (6.2)	246 (5.5)
	Strongly disagree	81 (18.5)	122 (21.7)	117 (22.0)	119 (23.4)	123 (16.8)	65 (16.8)	62 (13.1)	94 (19.8)	96 (25.0)	879 (19.6)
In the foreseeable future, all physicians will be replaced	Agree	14 (3.2)	33 (5.9)	30 (5.6)	27 (5.3)	49 (6.7)	31 (8.0)	35 (7.4)	32 (6.7)	25 (6.5)	276 (6.1)
	Disagree	210 (48.1)	238 (42.3)	163 (30.6)	185 (36.4)	315 (43.2)	168 (43.3)	201 (42.4)	213 (44.8)	135 (35.2)	1828 (40.7)
	Neutral	51 (11.7)	87 (15.5)	83 (15.6)	82 (16.1)	132 (18.1)	60 (15.5)	116 (24.5)	59 (12.4)	59 (15.4)	729 (16.2)
	Strongly agree	7 (1.6)	21 (3.7)	36 (6.8)	21 (4.1)	31 (4.2)	16 (4.1)	20 (4.2)	23 (4.8)	23 (6.0)	198 (4.4)
	Strongly disagree	155 (35.5)	184 (32.7)	221 (41.5)	193 (38.0)	203 (27.8)	113 (29.1)	102 (21.5)	148 (31.2)	142 (37.0)	1461 (32.5)
These developments frighten me	Agree	120 (27.5)	140 (24.9)	89 (16.7)	91 (17.9)	149 (20.4)	110 (28.4)	107 (22.6)	89 (18.7)	95 (24.7)	990 (22.0)
	Disagree	95 (21.7)	96 (17.1)	98 (18.4)	115 (22.6)	123 (16.8)	65 (16.8)	90 (19.0)	128 (26.9)	77 (20.1)	887 (19.7)
	Neutral	142 (32.5)	183 (32.5)	193 (36.2)	174 (34.3)	213 (29.2)	137 (35.3)	144 (30.4)	160 (33.7)	102 (26.6)	1448 (32.2)
	Strongly agree	45 (10.3)	100 (17.8)	91 (17.1)	71 (14.0)	198 (27.1)	43 (11.1)	105 (22.2)	53 (11.2)	74 (19.3)	780 (17.4)
	Strongly disagree	35 (8.0)	44 (7.8)	62 (11.6)	57 (11.2)	47 (6.4)	33 (8.5)	28 (5.9)	45 (9.5)	36 (9.4)	387 (8.6)
These developments make radiology more exciting to me	Agree	152 (34.8)	198 (35.2)	179 (33.6)	161 (31.7)	240 (32.9)	156 (40.2)	173 (36.5)	186 (39.2)	136 (35.4)	1581 (35.2)
	Disagree	59 (13.5)	55 (9.8)	46 (8.6)	50 (9.8)	68 (9.3)	42 (10.8)	45 (9.5)	35 (7.4)	43 (11.2)	443 (9.9)
	Neutral	126 (28.8)	176 (31.3)	167 (31.3)	170 (33.5)	217 (29.7)	115 (29.6)	144 (30.4)	135 (28.4)	107 (27.9)	1357 (30.2)
	Strongly agree	86 (19.7)	115 (20.4)	128 (24.0)	109 (21.5)	186 (25.5)	61 (15.7)	101 (21.3)	108 (22.7)	86 (22.4)	980 (21.8)
	Strongly disagree	14 (3.2)	19 (3.4)	13 (2.4)	18 (3.5)	19 (2.6)	14 (3.6)	11 (2.3)	11 (2.3)	12 (3.1)	131 (2.9)
These developments make medicine in general more exciting to me	Agree	149 (34.1)	225 (40.0)	187 (35.1)	164 (32.3)	262 (35.9)	165 (42.5)	179 (37.8)	216 (45.5)	163 (42.4)	1710 (38.1)
	Disagree	58 (13.3)	48 (8.5)	40 (7.5)	42 (8.3)	59 (8.1)	37 (9.5)	37 (7.8)	33 (6.9)	43 (11.2)	397 (8.8)
	Neutral	127 (29.1)	156 (27.7)	117 (22.0)	159 (31.3)	180 (24.7)	98 (25.3)	115 (24.3)	105 (22.1)	92 (24.0)	1149 (25.6)

	Strongly agree	86 (19.7)	121 (21.5)	179 (33.6)	129 (25.4)	209 (28.6)	75 (19.3)	130 (27.4)	110 (23.2)	76 (19.8)	1115 (24.8)
	Strongly disagree	17 (3.9)	13 (2.3)	10 (1.9)	14 (2.8)	20 (2.7)	13 (3.4)	13 (2.7)	11 (2.3)	10 (2.6)	121 (2.7)
Artificial intelligence will never make the human physician expendable	Agree	148 (33.9)	152 (27.0)	139 (26.1)	134 (26.4)	202 (27.7)	129 (33.2)	120 (25.3)	154 (32.4)	96 (25.0)	1274 (28.4)
	Disagree	26 (5.9)	50 (8.9)	52 (9.8)	37 (7.3)	53 (7.3)	35 (9.0)	43 (9.1)	23 (4.8)	41 (10.7)	360 (8.0)
	Neutral	123 (28.1)	125 (22.2)	108 (20.3)	119 (23.4)	156 (21.4)	75 (19.3)	99 (20.9)	109 (22.9)	72 (18.8)	986 (22.0)
	Strongly agree	131 (30.0)	213 (37.8)	221 (41.5)	208 (40.9)	304 (41.6)	143 (36.9)	206 (43.5)	179 (37.7)	157 (40.9)	1762 (39.2)
	Strongly disagree	9 (2.1)	23 (4.1)	13 (2.4)	10 (2.0)	15 (2.1)	6 (1.5)	6 (1.3)	10 (2.1)	18 (4.7)	110 (2.4)
The impact of artificial intelligence alone will reduce the number of radiologists that are needed	Agree	185 (42.3)	236 (41.9)	167 (31.3)	176 (34.6)	231 (31.6)	158 (40.7)	157 (33.1)	160 (33.7)	150 (39.1)	1620 (36.1)
	Disagree	53 (12.1)	74 (13.1)	70 (13.1)	76 (15.0)	122 (16.7)	49 (12.6)	80 (16.9)	71 (14.9)	44 (11.5)	639 (14.2)
	Neutral	134 (30.7)	172 (30.6)	215 (40.3)	176 (34.6)	235 (32.2)	142 (36.6)	145 (30.6)	172 (36.2)	115 (29.9)	1506 (33.5)
	Strongly agree	59 (13.5)	62 (11.0)	66 (12.4)	63 (12.4)	100 (13.7)	31 (8.0)	85 (17.9)	54 (11.4)	54 (14.1)	574 (12.8)
	Strongly disagree	6 (1.4)	19 (3.4)	15 (2.8)	17 (3.3)	42 (5.8)	8 (2.1)	7 (1.5)	18 (3.8)	21 (5.5)	153 (3.4)
Artificial intelligence will improve radiology	Agree	177 (40.5)	271 (48.1)	233 (43.7)	204 (40.2)	287 (39.3)	197 (50.8)	214 (45.1)	223 (46.9)	171 (44.5)	1977 (44.0)
	Disagree	10 (2.3)	10 (1.8)	26 (4.9)	16 (3.1)	50 (6.8)	17 (4.4)	42 (8.9)	31 (6.5)	29 (7.6)	231 (5.1)
	Neutral	44 (10.1)	94 (16.7)	130 (24.4)	120 (23.6)	154 (21.1)	74 (19.1)	85 (17.9)	103 (21.7)	75 (19.5)	879 (19.6)
	Strongly agree	204 (46.7)	179 (31.8)	138 (25.9)	162 (31.9)	226 (31.0)	97 (25.0)	127 (26.8)	111 (23.4)	102 (26.6)	1346 (30.0)
	Strongly disagree	2 (0.5)	9 (1.6)	6 (1.1)	6 (1.2)	13 (1.8)	3 (0.8)	6 (1.3)	7 (1.5)	7 (1.8)	59 (1.3)
Artificial intelligence will improve medicine in general	Agree	201 (46.0)	266 (47.2)	221 (41.5)	238 (46.9)	299 (41.0)	209 (53.9)	207 (43.7)	235 (49.5)	176 (45.8)	2052 (45.7)
	Disagree	7 (1.6)	19 (3.4)	22 (4.1)	6 (1.2)	36 (4.9)	15 (3.9)	29 (6.1)	22 (4.6)	18 (4.7)	174 (3.9)
	Neutral	45 (10.3)	91 (16.2)	85 (15.9)	85 (16.7)	117 (16.0)	51 (13.1)	73 (15.4)	75 (15.8)	65 (16.9)	687 (15.3)

	Strongly agree	179 (41.0)	182 (32.3)	201 (37.7)	178 (35.0)	264 (36.2)	112 (28.9)	158 (33.3)	140 (29.5)	121 (31.5)	1535 (34.2)
	Strongly disagree	5 (1.1)	5 (0.9)	4 (0.8)	1 (0.2)	14 (1.9)	1 (0.3)	7 (1.5)	3 (0.6)	4 (1.0)	44 (1.0)
I am less likely to consider a career in radiology given the advancement of artificial intelligence	Agree	75 (17.2)	113 (20.1)	86 (16.1)	85 (16.7)	112 (15.3)	67 (17.3)	97 (20.5)	75 (15.8)	83 (21.6)	793 (17.7)
	Disagree	94 (21.5)	113 (20.1)	119 (22.3)	115 (22.6)	207 (28.4)	112 (28.9)	128 (27.0)	134 (28.2)	93 (24.2)	1115 (24.8)
	Neutral	198 (45.3)	228 (40.5)	207 (38.8)	208 (40.9)	271 (37.1)	154 (39.7)	158 (33.3)	179 (37.7)	135 (35.2)	1738 (38.7)
	Strongly agree	40 (9.2)	64 (11.4)	69 (12.9)	62 (12.2)	84 (11.5)	22 (5.7)	62 (13.1)	47 (9.9)	41 (10.7)	491 (10.9)
	Strongly disagree	30 (6.9)	45 (8.0)	52 (9.8)	38 (7.5)	56 (7.7)	33 (8.5)	29 (6.1)	40 (8.4)	32 (8.3)	355 (7.9)

Data are presented as frequency (%)

Table S3. Students' attitude towards artificial intelligence and medical curriculum

		Algeria	Egypt	Iraq	Jordan	Libya	Palestine	Sudan	Syria	Yemen	Total
All medical students should receive teaching in artificial intelligence	Strongly disagree	8 (1.8)	14 (2.5)	18 (3.4)	19 (3.7)	34 (4.7)	7 (1.8)	29 (6.1)	12 (2.5)	22 (5.7)	163 (3.6)
	Disagree	17 (3.9)	45 (8.0)	24 (4.5)	45 (8.9)	33 (4.5)	18 (4.6)	20 (4.2)	45 (9.5)	30 (7.8)	277 (6.2)
	Neutral	86 (19.7)	118 (21.0)	94 (17.6)	107 (21.1)	131 (17.9)	100 (25.8)	98 (20.7)	112 (23.6)	74 (19.3)	920 (20.5)
	Agree	95 (21.7)	192 (34.1)	126 (23.6)	149 (29.3)	163 (22.3)	137 (35.3)	87 (18.4)	131 (27.6)	102 (26.6)	1182 (26.3)
	Strongly agree	231 (52.9)	194 (34.5)	271 (50.8)	188 (37.0)	369 (50.5)	126 (32.5)	240 (50.6)	175 (36.8)	156 (40.6)	1950 (43.4)
Teaching in artificial intelligence will be beneficial for my career	Strongly disagree	7 (1.6)	10 (1.8)	15 (2.8)	10 (2.0)	19 (2.6)	5 (1.3)	22 (4.6)	13 (2.7)	17 (4.4)	118 (2.6)
	Disagree	10 (2.3)	38 (6.7)	22 (4.1)	37 (7.3)	38 (5.2)	16 (4.1)	31 (6.5)	36 (7.6)	34 (8.9)	262 (5.8)
	Neutral	75 (17.2)	143 (25.4)	118 (22.1)	110 (21.7)	139 (19.0)	91 (23.5)	74 (15.6)	107 (22.5)	72 (18.8)	929 (20.7)
	Agree	128 (29.3)	186 (33.0)	137 (25.7)	169 (33.3)	183 (25.1)	141 (36.3)	112 (23.6)	158 (33.3)	119 (31.0)	1333 (29.7)
	Strongly agree	217 (49.7)	186 (33.0)	241 (45.2)	182 (35.8)	351 (48.1)	135 (34.8)	235 (49.6)	161 (33.9)	142 (37.0)	1850 (41.2)
At the end of my medical degree, I will be confident in using basic healthcare artificial intelligence tools if required	Strongly disagree	112 (25.6)	56 (9.9)	29 (5.4)	36 (7.1)	38 (5.2)	17 (4.4)	29 (6.1)	67 (14.1)	25 (6.5)	409 (9.1)
	Disagree	78 (17.8)	100 (17.8)	57 (10.7)	76 (15.0)	70 (9.6)	54 (13.9)	53 (11.2)	73 (15.4)	56 (14.6)	617 (13.7)
	Neutral	99 (22.7)	162 (28.8)	163 (30.6)	164 (32.3)	191 (26.2)	116 (29.9)	125 (26.4)	143 (30.1)	133 (34.6)	1296 (28.9)
	Agree	74 (16.9)	118 (21.0)	118 (22.1)	131 (25.8)	184 (25.2)	125 (32.2)	95 (20.0)	100 (21.1)	92 (24.0)	1037 (23.1)
	Strongly agree	74 (16.9)	127 (22.6)	166 (31.1)	101 (19.9)	247 (33.8)	76 (19.6)	172 (36.3)	92 (19.4)	78 (20.3)	1133 (25.2)
At the end of my medical degree, I will have a better	Strongly disagree	122 (27.9)	76 (13.5)	46 (8.6)	48 (9.4)	50 (6.8)	20 (5.2)	39 (8.2)	71 (14.9)	35 (9.1)	507 (11.3)
	Disagree	76 (17.4)	112 (19.9)	69 (12.9)	90 (17.7)	98 (13.4)	64 (16.5)	67 (14.1)	81 (17.1)	79 (20.6)	736 (16.4)

understanding of the methods used to assess healthcare artificial intelligence algorithm performance	Neutral	115 (26.3)	180 (32.0)	154 (28.9)	178 (35.0)	229 (31.4)	136 (35.1)	134 (28.3)	155 (32.6)	115 (29.9)	1396 (31.1)
	Agree	58 (13.3)	110 (19.5)	135 (25.3)	109 (21.5)	177 (24.2)	110 (28.4)	100 (21.1)	97 (20.4)	98 (25.5)	994 (22.1)
	Strongly agree	66 (15.1)	85 (15.1)	129 (24.2)	83 (16.3)	176 (24.1)	58 (14.9)	134 (28.3)	71 (14.9)	57 (14.8)	859 (19.1)
Overall, at the end of my medical degree, I feel I will possess the knowledge needed to work with artificial intelligence in routine clinical practice	Strongly disagree	135 (30.9)	81 (14.4)	44 (8.3)	41 (8.1)	55 (7.5)	19 (4.9)	39 (8.2)	72 (15.2)	33 (8.6)	519 (11.6)
	Disagree	89 (20.4)	108 (19.2)	73 (13.7)	94 (18.5)	83 (11.4)	70 (18.0)	66 (13.9)	92 (19.4)	64 (16.7)	739 (16.5)
	Neutral	101 (23.1)	166 (29.5)	153 (28.7)	181 (35.6)	204 (27.9)	129 (33.2)	134 (28.3)	143 (30.1)	131 (34.1)	1342 (29.9)
	Agree	52 (11.9)	124 (22.0)	123 (23.1)	112 (22.0)	187 (25.6)	102 (26.3)	91 (19.2)	98 (20.6)	90 (23.4)	979 (21.8)
	Strongly agree	60 (13.7)	84 (14.9)	140 (26.3)	80 (15.7)	201 (27.5)	68 (17.5)	144 (30.4)	70 (14.7)	66 (17.2)	913 (20.3)

Data are presented as frequency (%)

Questionnaire in Arabic

معرفة وموقف وإدراك طلاب الطب العرب تجاه الذكاء الاصطناعي في الطب والأشعة: دراسة مقطعية متعددة الجنسيات

من خلال استكمال الاستبيان، فإنك توافق على ما يلي:

شكرا لك على المشاركة!

نحن مجموعة من الباحثين من الدول العربية نحاول فهم معرفة وموقف وإدراك وممارسة طلاب الطب تجاه الذكاء الاصطناعي وتطبيقاته في الطب بشكل عام وطب الأشعة على وجه التحديد.

سيستغرق هذا الاستبيان حوالي 10 دقائق لإكماله.

سيتم تخزين ردودك بشكل آمن. سيتمكن الباحثون الرئيسيون فقط في الدراسة من رؤية ردودك.

ونهدف إلى نشر النتائج التي سنحصل عليها في مؤتمر أكاديمي ومجلة أكاديمية مرموقة.

لأية استفسارات، يرجى الاتصال بنا على:
ahmedhafez21@med.menofia.edu.eg

- هذا الاستبيان يستهدف طلاب الطب فقط. إذا لم تكن طالبًا في كلية الطب، فالرجاء عدم المتابعة.

- لا يمكن ملء هذا الاستبيان إلا مرة واحدة. لذا إذا كنت قد ملأت هذا الاستبيان من قبل، فيرجى عدم المتابعة.

- يرجى محاولة تقديم إجابات صادقة بقدر المستطاع. لا تبحث في جوجل عن الإجابات التي لا تعرفها ولا تخمنها. ما عليك سوى اختيار ما تعرفه، وإذا كنت لا تعرف الإجابة، فما عليك سوى اختيار "لا أعرف".

الخصائص الفردية

- الجنس
(ذكر – أنثى)

- الدولة
(اليمن – الأردن – سوريا – فلسطين – العراق – مصر – المغرب – ليبيا – الجزائر – السودان - السعودية)

- الجامعة
(خاصة – حكومية – دولية)

- مكان المعيشة
(المدينة – الريف)

- السنة الدراسية
(الأولى – الثانية – الثالثة – الرابعة – الخامسة – السادسة – امتياز)

- هل تعتبر نفسك خبيرًا في التعامل مع التكنولوجيا خاصة الكمبيوتر؟
(موافق بشدة - موافق – محايد - غير موافق - غير موافق بشدة)

المعرفة

يرجى تقييم موافقتك على الأسئلة التالية:

- لدي فهم للمبادئ الحسابية الأساسية الخاصة بالذكاء الاصطناعي
(موافق بشدة - موافق – محايد - غير موافق - غير موافق بشدة)

- أنا على دراية بالمصطلحات المتعلقة بالذكاء الاصطناعي
(موافق بشدة - موافق – محايد - غير موافق - غير موافق بشدة)

- لدي فهم لقيود الذكاء الاصطناعي
(موافق بشدة - موافق – محايد - غير موافق - غير موافق بشدة)

الذكاء الاصطناعي والتعلم العميق في طب الأشعة

يتم حاليًا مناقشة "التعلم العميق" و "الذكاء الاصطناعي" على نطاق واسع في مجتمع طب الأشعة.

- هل كنت بالفعل على علم بهذه الموضوعات في مجال طب الأشعة؟
(نعم / لا)

- هل لديك فهم أساسي للتقنيات المستخدمة في هذه المواضيع؟
(نعم / لا)

- معرفتك وانخراطك في مجال الذكاء الاصطناعي تشمل: (يرجى تحديد كل ما ينطبق):
  - دورات في الذكاء الاصطناعي والتعلم الآلي
  - مشاريع علوم الحاسب التي تتضمن الذكاء الاصطناعي
  - أبحاث طب الأشعة التي تتضمن الذكاء الاصطناعي
  - لا شيء مما سبق

تختبر الأسئلة التالية مدى فهمك للتعلم العميق، وهو مجال فرعي من الذكاء الاصطناعي.
(الرجاء اختيار "لا أعرف" إذا كنت غير متأكد من معرفتك)

1. التعلم العميق هو مجال من مجالات التعلم الآلي التي تستخدم طبقات متعددة من الشبكات العصبية.
(صحيح – خطأ – لا اعرف)

2. تتعلم طرق التعلم العميق مباشرة من البيانات، دون الحاجة إلى استخراج الميزات المصممة يدويًا.
(صحيح – خطأ – لا اعرف)

3. يتطلب تطبيق التعلم العميق في مجال طب الأشعة قواعد بيانات كبيرة من الصور الطبية المصنفة.
(صحيح – خطأ – لا اعرف)

4. غالبًا ما تكون أنظمة التعلم العميق مبهمة: قد يكون من الصعب تحديد "عملية التفكير" الأساسية.
(صحيح – خطأ – لا اعرف)

السلوكيات

شعورك وموقفك تجاه الذكاء الاصطناعي والتعلم العميق في الطب عمومًا وطب الأشعة تحديدًا

برأيك الشخصي ما مدى دقة العبارات التالية؟

- الذكاء الاصطناعي سيحدث ثورة في طب الأشعة.
(موافق بشدة - موافق – محايد - غير موافق - غير موافق بشدة)

- الذكاء الاصطناعي سيحدث ثورة في الطب بشكل عام.
(موافق بشدة - موافق – محايد - غير موافق - غير موافق بشدة)

- سيتم استبدال أخصائيين طب الأشعة في المستقبل القريب.
(موافق بشدة - موافق – محايد - غير موافق - غير موافق بشدة)

- سيتم استبدال الطبيب البشري (الباطني) في المستقبل القريب.
(موافق بشدة - موافق – محايد - غير موافق - غير موافق بشدة)

- في المستقبل القريب، سيتم استبدال جميع الأطباء.
(موافق بشدة - موافق – محايد - غير موافق - غير موافق بشدة)

- هذه التطورات تخيفني.
(موافق بشدة - موافق – محايد - غير موافق - غير موافق بشدة)

- هذه التطورات تجعل طب الأشعة أكثر إثارة بالنسبة لي.
(موافق بشدة - موافق – محايد - غير موافق - غير موافق بشدة)

- تجعل هذه التطورات الطب بشكل عام أكثر إثارة بالنسبة لي.
(موافق بشدة - موافق – محايد - غير موافق - غير موافق بشدة)

- الذكاء الاصطناعي لن يجعل الطبيب البشري قابلًا للاستبدال أبدًا.
(موافق بشدة - موافق – محايد - غير موافق - غير موافق بشدة)

- إن تأثير الذكاء الاصطناعي وحده سيقلل من عدد أخصائيين طب الأشعة اللازمين.
(موافق بشدة - موافق – محايد - غير موافق - غير موافق بشدة)

- الذكاء الاصطناعي سوف يحسن طب الأشعة بشكل خاص.
(موافق بشدة - موافق – محايد - غير موافق - غير موافق بشدة)

- الذكاء الاصطناعي سيحسن الطب بشكل عام.
(موافق بشدة - موافق – محايد - غير موافق - غير موافق بشدة)

- من غير المحتمل أن أفكر في الحصول على وظيفة في تخصص طب الأشعة نظرًا لتقدم الذكاء الاصطناعي.
(موافق بشدة - موافق – محايد - غير موافق - غير موافق بشدة)

الذكاء الاصطناعي والمناهج الطبية

- يجب أن يتلقى جميع طلاب الطب تعليمًا في الذكاء الاصطناعي
(موافق بشدة - موافق – محايد - غير موافق - غير موافق بشدة)

- تدريس الذكاء الاصطناعي سيكون مفيدًا لحياتي المهنية
(موافق بشدة - موافق – محايد - غير موافق - غير موافق بشدة)

- في نهاية دراستي للطب، سأكون واثقًا من قدرتي على استخدام أدوات الذكاء الاصطناعي الخاصة بالرعاية الصحية الأساسية إذا لزم الأمر
(موافق بشدة - موافق – محايد - غير موافق - غير موافق بشدة)

- في نهاية دراستي للطب، سيكون لدي فهم أفضل للطرق المستخدمة لتقييم أداء خوارزميات الذكاء الاصطناعي في الرعاية الصحية
(موافق بشدة - موافق – محايد - غير موافق - غير موافق بشدة)

- بشكل عام، في نهاية دراستي للطب، أشعر أنني سأمتلك المعرفة اللازمة للعمل مع الذكاء الاصطناعي في الممارسة الطبية الروتينية
(موافق بشدة - موافق – محايد - غير موافق - غير موافق بشدة)

الإدراك

- هل يمكنك أن تأخذ في الاعتبار استخدام هذا التسلسل في عملك كطبيب؟
الصور السريرية للمرضى تخضع لتحليل الذكاء الاصطناعي ثم يقوم أحد المتخصصين بعد ذلك بمراجعة كل من الصور ونتائج الذكاء الاصطناعي.
(نعم – لا – غير متأكد)

تطبيقات الذكاء الاصطناعي في طب الأشعة

- ما هي التطبيقات المحتملة للذكاء الاصطناعي في الأشعة التي تراها؟

1. الكشف الآلي عن الأمراض من خلال فحوصات التصوير
(موافق بشدة - موافق – محايد - غير موافق - غير موافق بشدة)

2. التشخيص الآلي من خلال فحوصات التصوير
(موافق بشدة - موافق – محايد - غير موافق - غير موافق بشدة)

3. التوجيه الآلي لاختبارات التصوير المناسبة
(موافق بشدة - موافق – محايد - غير موافق - غير موافق بشدة)

التدريس أو التدريب في مجال الذكاء الاصطناعي

- هل تلقيت تعليمًا أو تدريبًا في مجال الذكاء الاصطناعي؟
(نعم / لا)

إذا أجبت بنعم على السؤال السابق،

- فهل كان هذا التدريس أو التدريب جزءًا إلزاميًا من شهادتك الطبية؟
(نعم / لا)

- فيرجى تقييم فائدة التدريس أو التدريب الذي تلقيته
(مفيد للغاية - مفيد جدًا - مفيد إلى حد ما - غير مفيد للغاية - غير مفيد على الإطلاق)

التخصصات

- أي من هذه التخصصات تعتقد أنه سيتأثر في أقرب وقت ممكن بالذكاء الاصطناعي؟
(الجراحة - الطب الباطني - علم الأمراض - الأشعة التشخيصية - الأمراض الجلدية - طب الأسرة - الأورام - أخرى: ....)

Questionnaire in English

By completing the questionnaire, you consent to the below:

Thank you for taking part!

We are a group of researchers from the Arab countries trying to better understand the knowledge, attitude, perception, and practice of undergraduate medical students towards artificial intelligence (AI) and its implementation in medicine generally and radiology specifically.

This survey will approximately 10 mins to complete.

Your responses will be stored securely. Only the principal investigators of the study will be able to view your responses.

We aim to present the aggregate results at an academic conference and an academic journal.

For any queries, please contact us at:
ahmedhafez21@med.menofia.edu.eg

- This survey only targets medical students. If you are not a medical student, please don't continue.

- This questionnaire can only be filled out once. So, if you filled this questionnaire before, please don't continue.

- Please try to provide honest answers as much as you can. Don't search google for answers you don't know and don't guess. Just choose what you know and if you don't know the answer, simply choose I don't know.

Demographics

- Gender
  (Male – Female)

- Country of residence
  (Egypt – Algeria – Iraq – Jordan – Libya – Palestine– Sudan – Syria – Yemen)

- University
  (Private – Governmental – International)

- Where do you live?
  (Urban – Rural)

- Grade
  (1 – 2 – 3 – 4 – 5 – 6 – Intern or Emtiaz)

- I consider myself a tech-savvy (well informed about or proficient in the use of modern technology, especially computers)
  (Strongly agree - Agree - Neutral - Disagree - Strongly disagree)

Knowledge

Please rate your agreement to the following questions:

- I have an understanding of the basic computational principles of artificial intelligence

(Strongly agree - Agree - Neutral - Disagree - Strongly disagree)

- I am comfortable with the nomenclature related to artificial intelligence

(Strongly agree - Agree - Neutral - Disagree - Strongly disagree)

- I have an understanding of the limitations of artificial intelligence

(Strongly agree - Agree - Neutral - Disagree - Strongly disagree)

AI and deep learning as a topic in radiology

'Deep Learning' and 'Artificial Intelligence' are currently being broadly discussed in the radiological community.

- Were you already aware of these topics in radiology?
  (Yes – No)
- Do you personally have a basic understanding of the technologies used in these topics?
  (Yes – No)

- Your exposure to artificial intelligence includes: (Please select all that apply)

- Courses on artificial intelligence/machine learning
- Computer science projects involving artificial intelligence
- Radiology research involving artificial intelligence
- None of the above

The following questions test your understanding of deep learning, a subtype of artificial intelligence. (Please choose "I don't know" if you are unsure about the statement.)

1. Deep learning is a class of machine learning algorithms that use multiple layers of neural networks.
   (True – False - I don't know)

2. Deep learning methods learn directly from data, without the need for manual feature extraction.
   (True – False - I don't know)

3. The application of deep learning in radiology requires large databases of labeled medical images.
   (True – False - I don't know)

4. Deep learning systems are often opaque: it can be difficult to delineate the underlying "thought process".
   (True – False - I don't know)

Attitude

Feelings and attitudes towards AI and deep learning in medicine and radiology

In your personal opinion, how accurate are the following statements?

1. Artificial intelligence will revolutionize radiology.
   (Strongly agree - Agree - Neutral - Disagree - Strongly disagree)

2. Artificial intelligence will revolutionize medicine in general.
   (Strongly agree - Agree - Neutral - Disagree - Strongly disagree)

3. The human radiologist will be replaced in the foreseeable future.
   (Strongly agree - Agree - Neutral - Disagree - Strongly disagree)

4. The human (non-interventional) physician will be replaced foreseeable future.
   (Strongly agree - Agree - Neutral - Disagree - Strongly disagree)

5. In the foreseeable future, all physicians will be replaced.
   (Strongly agree - Agree - Neutral - Disagree - Strongly disagree)

6. These developments frighten me.
   (Strongly agree - Agree - Neutral - Disagree - Strongly disagree)

7. These developments make radiology more exciting to me.
   (Strongly agree - Agree - Neutral - Disagree - Strongly disagree)

8. These developments make medicine in general more exciting to me.
   (Strongly agree - Agree - Neutral - Disagree - Strongly disagree)

9. Artificial intelligence will never make the human physician expendable.
   (Strongly agree - Agree - Neutral - Disagree - Strongly disagree)

10. The impact of artificial intelligence alone will reduce the number of radiologists that are needed.
    (Strongly agree - Agree - Neutral - Disagree - Strongly disagree)

11. Artificial intelligence will improve radiology.
    (Strongly agree - Agree - Neutral - Disagree - Strongly disagree)

12. Artificial intelligence will improve medicine in general.
    (Strongly agree - Agree - Neutral - Disagree - Strongly disagree)

13. I am ***LESS*** likely to consider a career in radiology, given the advancement of AI.
    (Strongly agree - Agree - Neutral - Disagree - Strongly disagree)

AI and medical curriculum

- All medical students should receive teaching in artificial intelligence
  (Strongly agree - Agree - Neutral - Disagree - Strongly disagree)

- Teaching in artificial intelligence will be beneficial for my career
  (Strongly agree - Agree - Neutral - Disagree - Strongly disagree)

- At the end of my medical degree, I will be confident in using basic healthcare AI tools if required
  (Strongly agree - Agree - Neutral - Disagree - Strongly disagree)

- At the end of my medical degree, I will have a better understanding of the methods used to assess healthcare AI algorithm performance
  (Strongly agree - Agree - Neutral - Disagree - Strongly disagree)

- Overall, at the end of my medical degree, I feel I will possess the knowledge needed to work with AI in routine clinical practice
  (Strongly agree - Agree - Neutral - Disagree - Strongly disagree)

Perception

- Would you consider using the following clinical workflow? Patients' clinical images undergo artificial intelligence analysis. A specialist subsequently reviews both the image and the artificial intelligence findings.
  - Yes
  - No
  - Unsure

Applications for AI in radiology

What potential applications for AI in radiology do you see?

- Automated detection of pathologies in imaging exams
  (Strongly agree - Agree - Neutral- Disagree - Strongly disagree)

- Automated diagnosis in imaging exams
  (Strongly agree - Agree - Neutral- Disagree - Strongly disagree)

- Automated indication of appropriate imaging exams
  (Strongly agree - Agree - Neutral- Disagree - Strongly disagree)

AI training

- I have received teaching/training in artificial intelligence
  (Yes – No)

  If you have answered yes to the previous question,

- Was this teaching/training a compulsory part of your medical degree?
  (Yes – No)

- Please rate the usefulness of the teaching/training you have received
  (Extremely useful - Very useful - Somewhat useful - Not so useful - Not at all useful)

Specialties affected

Which of these specialties do you think will be impacted the earliest and most?
(Surgery – internal medicine – pathology – diagnostic radiology – dermatology – family practice – oncology – other…..)
